# Functional characterization of oxazolone-induced colitis and survival improvement by vagus nerve stimulation

**DOI:** 10.1371/journal.pone.0197487

**Published:** 2018-05-23

**Authors:** Elisa Meroni, Nathalie Stakenborg, Pedro J. Gomez-Pinilla, Gert De Hertogh, Gera Goverse, Gianluca Matteoli, Simon Verheijden, Guy E. Boeckxstaens

**Affiliations:** 1 KU Leuven, Department of Chronic Diseases, Metabolism and Ageing, Translational Research Center for Gastrointestinal Disorders (TARGID), Leuven, Belgium; 2 KU Leuven, Department of Imaging and Pathology, Translational Cell and Tissue Research, Leuven, Belgium; University of California Los Angeles, UNITED STATES

## Abstract

**Background:**

Oxazolone-induced colitis has been frequently used in literature as a model of IBD, but insights into the underlying immune response and pathological features are surprisingly still very limited. Vagus nerve stimulation (VNS) has proven to be effective in innate and Th1/Th17 predominant inflammatory models, including pre-clinical models of colitis, however to what extent VNS is also effective in ameliorating Th2-driven colitis remains to be studied. In the present study, we therefore further characterized the immune response in oxazolone-induced colitis and investigated the potential therapeutic effect of VNS.

**Methods:**

Colitis was induced in Balb/c mice by cutaneous sensitization with 3% oxazolone followed by intracolonic administration of 1% oxazolone 7 days later. To evaluate the effect of VNS on the development of Th2-driven colitis, VNS and sham-treated mice were challenged with 1% oxazolone.

**Results:**

Intracolonic oxazolone administration resulted in a severe destruction of the colonic mucosa and a rapid drop in body temperature leading to a 65% mortality rate at day 5. Severe infiltration of neutrophils and monocytes was detected 6h after oxazolone administration which was associated with a Th2-type inflammatory response. VNS significantly improved survival rate which correlated with decreased levels of HMGB1 and reduced colonic (*il6* and *cxcl1* mRNA) and serum cytokine levels (IL-6, TNFα and CXCL1) compared to sham treated mice.

**Conclusions:**

Oxazolone-induced colitis rather represents a model of sepsis and, at best, may resemble a severe type of ulcerative colitis, associated with early and severe mucosal damage and a high mortality rate. VNS reduces colonic inflammation and improves survival in this model, supporting the anti-inflammatory properties of VNS, even in an aggressive model as oxazolone-induced colitis.

## Introduction

Approximately 2 million people worldwide suffer from inflammatory bowel disease (IBD), comprising Crohn’s disease (CD) and ulcerative colitis (UC) [1]. IBD is a multifactorial disorder characterized by chronic inflammation of the gastrointestinal (GI) tract resulting from an inappropriate innate and/or adaptive immune response towards the microbiome [2]. In the past few decades, many efforts have been undertaken to improve the treatment of IBD with the introduction of biologicals, such as TNFα antibodies. Nevertheless, mainly due to its multifactorial nature, not all patients benefit from the currently available therapeutics, warranting further preclinical and clinical efforts to optimize treatment of IBD [3].

Novel treatments should mainly focus on sustained reprogramming of the aberrant and chronic immune response in the GI tract. One of the therapies currently under investigation for the treatment of CD is chronic vagus nerve stimulation (VNS). Recent studies indeed demonstrated that pharmacological and electrical activation of the vagus nerve improves clinical and inflammatory parameters in rodent models of IBD, i.e. DSS-induced colitis and TNBS colitis [4] [5]. These studies have led to the initiation of clinical pilot studies evaluating the therapeutic efficacy in CD patients [6] [7]. However, both DSS-induced colitis and TNBS-induced colitis do not fully recapitulate the complex etiopathogenesis of IBD. Acute exposure to the surfactant DSS leads to disruption of the epithelial barrier and subsequent activation and infiltration of innate immune cells in the intestinal mucosa, making it a valid model to study the involvement of myeloid cells in the development of the disease [8]. TNBS is considered to represent a proper model to study aberrant type 1 helper T (Th1) cells responses, as intracolonic exposure to the haptenating agent TNBS typically leads to acute activation Th1 cells [9]. Of note, chronic activation of type 2 (Th2) immune responses is proposed to be the main driver of disease, in particular in UC [10]. To what extent VNS may be effective in Th2-driven colitis remains however unclear.

To date, oxazolone-induced colitis represents the most studied murine model of Th2-driven inflammation. In this model, colitis is induced in mouse strains susceptible to Th2 immune activation (SJL/J and Balb/c) by intracolonic instillation of the haptenating agent oxazolone dissolved in ethanol after a skin pre-sensitization step [11]. Notably, it has been shown that oxazolone colitis affects only the distal colon and particularly mucosal layers. In these mice, histological features and Th2 cytokine production (IL-4, IL-5 and IL-13) of unstimulated and αCD3/αCD28-stimulated lamina propria T cells are, at least in part, similar to characteristics observed in human UC [12]. Surprisingly though, only limited detailed information is available with regard to the time course and cytokine profile of the immune response involved in this model of colitis. In this study, we therefore further functionally characterized this model and subsequently investigated the therapeutic effect of VNS.

## Materials and methods

### Experimental animals and ethics statements

Animal studies were conducted in a gender- and age-matched manner. Eight to twelve week old wild-type female BALB/c mice were bred at the KU Leuven animal facility under environmentally controlled conditions (light on from 8:00 AM to 8:00 PM; 20°C-22°C, 55% humidity). Mice were housed with *ad libitum* access to standard rodent food and water. All animals were treated carefully in strict accordance with the ethical guidelines and all experimental procedures were approved by the Animal Care and Animal Experiments Committee of KU Leuven (P002/2014; P003/2014; Leuven, Belgium). The number of animals used per group was based on previous experimental results and observed variability. All surgeries were performed under anesthesia and all efforts were made to minimize suffering.

### Electrical stimulation of the vagus nerve

Anesthetized (2.5% isoflurane) (ISO-VET, Eurovet NV/SA, Heusden-Zolder, Belgium), non-implanted mice underwent surgery and VNS or Sham-stimulation.A ventral midline cervical incision was made between the mandible and sternum, subcutaneous tissue was dissected and retracted laterally. The mandibular salivary glands were bluntly separated and retracted laterally. The right cervical vagus nerve was isolated from the carotid artery and stimulated electrically using a bipolar platinum electrode (Bilaney, Dusseldorf, Germany). Continuous electrical stimuli consisted of square pulses (1 mA, 1 ms, 5 min duration) at 5 Hz (Keithley Instruments, Cleveland, Ohio) as previously described [13]. Sham-operated mice were handled similarly, but the vagus nerve was not dissected from the carotid artery to avoid mechanical stimulation. VNS was applied 15 min prior oxazolone (or ethanol). Mice were kept in heated cages immediately after the surgery until the effect of anesthesia was worn off in order to avoid hypothermia.

### Animal experiments

The immunological response of these mice was assessed at different time points 6 and 24h after colitis induction and survival proportion was monitored for 5 days after oxazolone administration.

Sixty BALB/c mice were previously randomized into five groups: Naïve (naïve, n = 10); EtOH-treated (50% EtOH, n = 10); oxazolone-treated (1% Oxa, n = 10); sham-treated/Oxa (Sham, n = 15); VNS-treated/Oxa (VNS, n = 15). Colitis in BALB/c mice was induced by oxazolone as described [14] [15]. Mice were sensitized by epicutaneous application of 3% oxazolone (4-ethoxymethylene-2-phenyl-2-oxazolin-5-one, Sigma-Aldrich, Saint Louis, Missouri) at a dilution of 4:1 in a mixture of acetone and olive oil (100 μl) on day 0, followed by intracolonic administration of 1% oxazolone dissolved in 50% ethanol (100 μl) (or 50% ethanol for control group EtOH-treated mice) on day 7 using an intravenous catheter (BD Insyte, 14GA, 2,1x45 mm) inserted 3 cm in the left colon. Mice were maintained in the head-down position for 2 min following intracolonic administration. Mice received VNS or sham stimulation for 5 min using standard stimulation parameters (1 mA, 1 ms, and 5 min duration). Naïve mice were left untreated.

### Quantification of disease activity

After oxazolone administration mice were monitored for body temperature every 30 minutes for 6 hours using Implantable Electronic ID Transponder (Bio Medic Data System Inc., Delaware). Mice that lost more than 20% of their initial body temperature were immediately euthanized with CO_2_ overdose. Extra attention was paid for visual clues like long-lasting pilo-erection and isolation from the group, so we could intervene in the event of a severe acute response to the hapten. Body weight of mice was measured daily, from day 0 to day 5. Mice that lost more than 20% of their initial body weight were immediately euthanized with CO_2_ overdose. The clinical disease activity index (DAI) was measured daily using the protocol previously described [16]. The scores from these three parameters were summed as the DAI, ranging from 0 (healthy) to 12 (maximal severity of colitis). All mice were sacrificed 6 or 24 hours after colitis induction for assessment of immunological responses or on experimental day 5 for survival proportion assessment. No mice reached the human endpoints.

### Histological examination

After sacrificing the mouse, 6 or 24 hours post oxazolone administration, the colon was removed and segments were immediately fixed in 4% buffered formalin (Merck, Darmstadt, Germany). After paraffin embedding, 5 μM thin sections were cut and stained with hematoxylin (Sigma-Aldrich, Saint Louis, Missouri) and eosin (Sigma-Aldrich, Saint Louis, Missouri) (H&E). Samples of colon were visualized with a microscope (BX 41 Olympus, Aartselaar, Belgium) connected to a camera (XM10 Olympus, Tokyo, Japan) and Cell^F software (Olympus, Tokyo, Japan) was used for tissue sampling. All stained sections were histologically examined by an experienced pathologist for evidence of colitis in a blinded fashion using a validated scoring system (Tables 1 and 2) [12] [17]. The total colonic score was calculated as the sum of the individual scores from the sections of colon with a maximum value of 12 or 15 depending on the scoring system as shown in Tables 1 or 2.

**Table 1:** Histological injury score (HIS).

Feature	0	1	2	3
Enterocyte loss	None	Mild	Moderate	Severe
Lamina propria mononuclear cells	None	Mild	Moderate	Severe
Neutrophils	None	Mild	Moderate	Severe
Epithelial hyperplasia	None	Mild	Moderate	Severe

**Table 2:** Histological assessment of colitis parameters (HAC).

Feature	0	1	2	3
Reduction in goblet cell number	None	Mild	Moderate	Severe
Ulceration	None	Mild	Moderate	Severe
Mononuclear cell infiltration	None	Mild	Moderate	Severe
Edema formation	None	Mild	Moderate	Severe
Apoptosis crypt epithelium	None	Mild	Moderate	Severe

### RNA extraction and gene expression

Total RNA was extracted from colonic tissue of oxazolone-treated mice. Tissues were homogenized with a TissueLyser II homogenizer (Qiagen, Hilden, Germany). RNA extraction was performed using RNeasy Mini Kit (Qiagen, Hilden, Germany) following the manufacturer’s instructions. Total RNA was retro-transcribed into cDNA using qScript cDNA SuperMix (Quanta Biosciences, Gaithersburg, Maryland) according to the manufacturer’s instructions, and gene expression was assayed by quantitative reverse transcription PCR (RT-PCR). Quantitative real-time transcription polymerase chain reactions were performed with the FastStart SYBR Green Master mix (Roche, Basel, Switzerland) using the LightCycler® 96 (Roche, Basel, Switzerland). Complementary DNA samples were assayed in duplicate and the expression levels of the genes of interest were normalized to the expression levels of the reference gene *rpl32*. The mean relative gene expression was calculated using the 2^-ΔΔCT^ method [18]. Primer sequences used are listed in Table 3.

**Table 3:** Primer sequences used for quantitative RT-PCR.

Gene	Sense	Antisense
***Rpl32***	5’-AAGCGAAACTGGCGGAAAC-3’	5’-TAACCGATGTTGGGCATCAG-3’
***Il4***	5’-GGCATTTTGAACGAGGTCACA-3’	5’-GACGTTTGGCACATCCATCTC-3’
***Il6***	5’-CCATAGCTACCTGGAGTACATG-3’	5’-TGGAAATTGGGGTAGGAAGGAC-3’
***Il9***	5′-ATGGTGAGCGGCCGCCACAAGGACAATGGGTTAGGGC-3′	5′-TGCTCCAACTAGTTCCTACCTATGCAACAC CGGG-3′
***Tnfa***	5’-TCTTCTCATTCCTGCTTGTGG-3’	5’-CACTTGGTGGTTTGCTACGA-3’
***Cxcl1***	5’- GCTGGGATTCACCTCAAGAA-3’	5’-TCTCCGTTACTTGGGGACAC-3’
***Il13***	5’- ATGGCCTCTGTAACCGCAAG– 3’	5’- GGCTGGAGACCTGTGAAACG -3’

### Isolation of splenocytes

Splenocytes were obtained from homogenized spleen in phosphate-buffered saline 1X (PBS) (Lonza, Verviers, Belgium) + 1% fetal bovine serum (FBS) (Nuaillé, France). Blood cells were lysed with lysis buffer (EDTA 68,5 μMol, NaHCO_3_ 5,95 mMol, NH_4_Cl 77,57 mMol dissolved in 500 ml of water) and cell suspensions were centrifuged at 4°C. Cells were then filtered with a 70 μm cell strainer and cultured at 37°C in RPMI medium + 10% FBS. Twelve hours later, supernatant was collected and cytometric bead array (CBA) analysis was performed.

### Immunofluorescence

Segments of spleen and colon were isolated and cryosections were prepared. Sections were post-fixed with cold acetone (2 min) and followed by pretreatment with Na Azide (0.1%) (Merck, Darmstadt, Germany) and H_2_O_2_ (0.3%) for 15 min. For immune-labeling experiments, tissues were blocked with 1% albumin from bovine serum (BSA) (ThermoFisher Scientific, Waltham, Massachusetts) dissolved in PBS for 1 hour. All primary and secondary antibodies were diluted in 1% BSA + 0.3% of Triton-X100 (ThermoFisher Scientific, Waltham, Massachusetts).The whole-mounts were incubated overnight at 4°C with the following primary antibodies: rabbit anti-Tyrosine Hydroxylase (TH) (1:1000, Millipore, Billerica, Massachusettes), rat anti-IL6 (1:250, ThermoFisher Scientific, Waltham, Massachusetts) and goat anti-TNFα (1:250, R&D Systems, Minneapolis, Minnesota). After washing in PBS, the whole-mounts were incubated for 1 hour at room temperature with the following fluorescently-labeled secondary antibodies (all from Jackson Immunoresearch, Bar Harbor, California): Cy3-conjugated donkey anti-rabbit IgG (1:1000), Cy3-conjugated anti-rat IgG (1:500) and Cy3-conjugated anti-goat IgG (1:500). Finally, samples were rinsed in PBS, mounted in SlowFade Diamond Antifade mountant (ThermoFischer Scientific, Waltham, Massachusetts) and evaluated using fluorescence microscopy (Cell^**^**^F software, Olympus, Tokyo, Japan). Images were acquired with XM10 camera (Olympus, Tokyo, Japan) using a 20X objective (443.8μm × 332.8μm).

### Cytometric bead array (CBA)

Quantification of IL-6, CXCL1 and TNFα was determined in serum samples and in supernatant of spleen samples of mice sacrificed at time 0, 6 and 24 hours post oxazolone administration and incubated for 12 hours at 37°C in RPMI complete medium using CBA according to the manufacturer’s instructions. Samples were acquired on FACSCanto HTS flow cytometer (BD Bioscience, Erembodegem-Dorp, Belgium) and analyzed by FCAP v3.0 analysis software (Soft Flow, Inc, Pecs, Hungary).

### Enzyme-linked immunosorbent assay (ELISA)

High-mobility group box (HMGB)-1 protein in plasma was quantified using an ELISA kit (LifeSpan BioSciences, Inc. Seattle, Washington) according to the manufacturer’s instructions.

### Isolation of leukocyte subpopulations and flow cytometry analysis

Cell suspensions were prepared from the colon *lamina propria* (LP) as previously described [19]. Live cells were identified using DAPI (ThermoFischer Scientific, Waltham, Massachusetts), and Fc receptors were blocked with an antibody for CD16/CD32 (BD Pharmigen, Franklin Lakes, New Jersey) for 10 min at room temperature. After incubation, the cells were stained at 4°C for 1 hour with labeled antibodies: CD11b (M1/70; BD Biosciences), SiglecF (1RNM44N, eBioscience), Ly6G (1A8, BD Biosciences), Ly6C (AL-21, BD Biosciences), MHCII (M5/114.15.2, eBioscience), CD45 (104, BD Bioscience), FceRIa (MAR-1, eBioscience), CD117 (2B8, eBioscience), CD49b (DX5, eBioscience), CD4 (RM4-5, eBioscience), CD3a (145-2C11, BD Biosciences), Ly49c (5E6, BD Bioscience). The intracellular expression of IL5 in CD4 T cells was analyzed using a Cytofix/Cytoperm Kit according to the manufacturer’s instructions (BD Bioscience, Erembodegem-Dorp, Belgium). In brief, lymphocytes were incubated with 50 ng/mL phorbol 12-myristate 13-acetate (PMA; Sigma-Aldrich, Saint Louis, Missouri), 500 ng/mL ionomycin (Sigma-Aldrich) and monensin (GolgiStop, BD Biosciences) in complete medium at 37° C for 4 hours. After surface staining with CD45 and CD4, cells were permeabilized and intracellular cytokine staining was performed using anti-IL5 mAb (4241824; eBioscience). Samples were then washed 2 times in PBS containing 5% FBS and analyzed immediately. Flow cytometric analysis was performed on a FACSCanto HTS flow cytometer (BD Bioscience, Franklin Lakes, New Jersey).

### Statistical analyses

Statistical analysis was performed with GraphPad Prism Software. The results are presented as the mean ± standard error of the mean (mean ± S.E.M.), a p value less than 0.05 was considered statistically significant. Survival rate is shown by Kaplan-Meier survival curves and statistical significance was determined with Gehan-Breslow-Wilcoxon test, a p value less than 0.05 was considered statistically significant.

## Results

### Oxazolone-induced colitis resulted in a high mortality rate and hypothermia

Oxazolone-induced colitis is generally described as a Th2-mediated model of colitis, but nevertheless it remains poorly characterized. For this reason, we decided to characterize in more depth the immune response in this colitis model.

**Fig 1:**
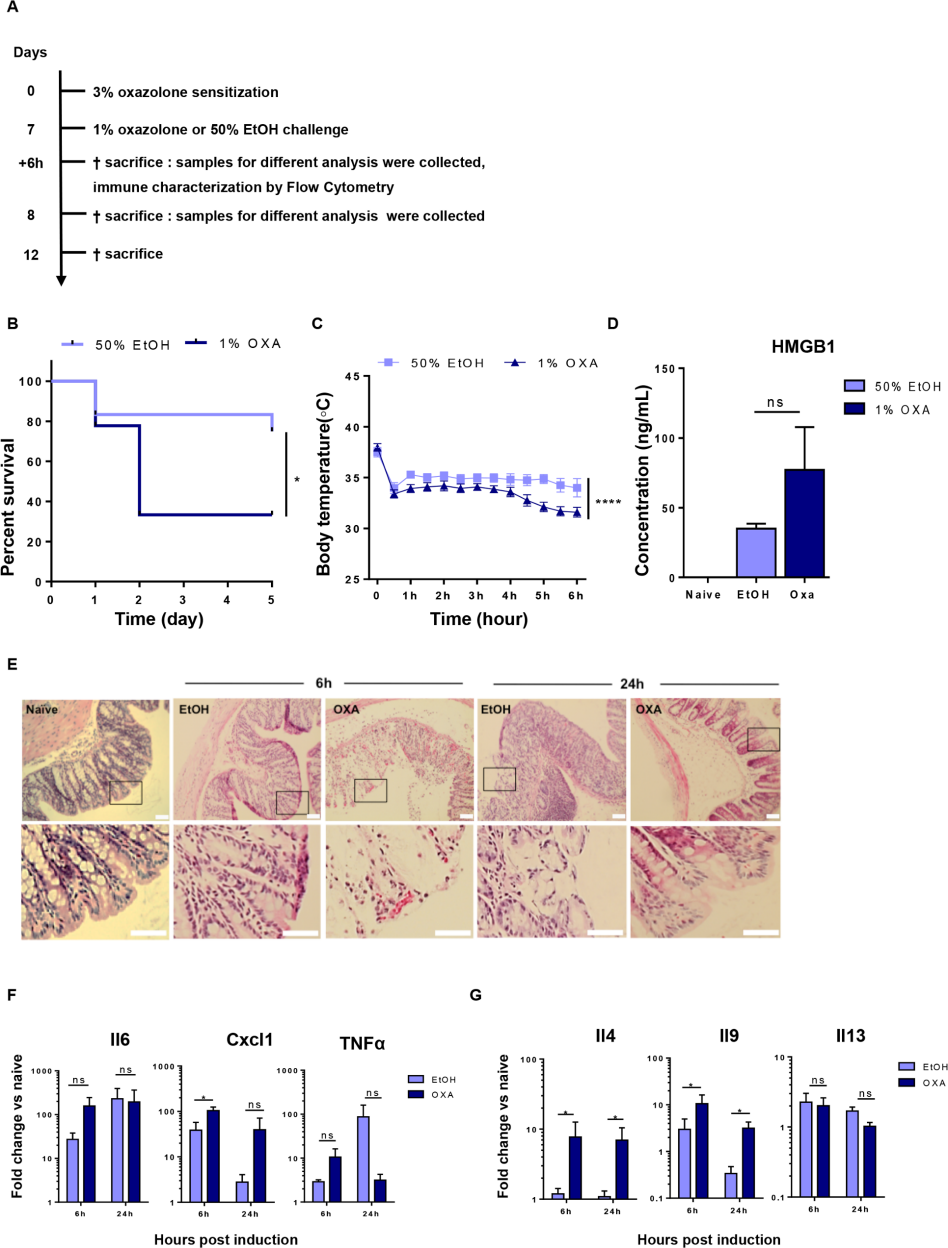
Characterization of oxazolone-induced colitis model. (A) Wild type BALB/c mice were sensitized by epicutaneous application of 3% oxazolone at a dilution of 4:1 in a mixture of acetone and olive oil (100**μ**l) on day 0, followed by intracolonic administration of 1% oxazolone dissolved in 50% ethanol (100μl) (or 50% ethanol for control group EtOH-treated mice) on day 7 using an intravenous catheter inserted 3 cm in the colon. Mice were sacrificed 6, 24 hours and 5 days post oxazolone administration and tissue samples were collected for different analysis. (B) Survival proportion was assessed daily. Mortality is expressed as survival rate and shown by Kaplan-Meier survival curves; statistical significance of Kaplan-Meier survival curves was determined with Gehan-Breslow-Wilcoxon test. *p < 0.05; (n = 12–18 mice per group). (C) Repeated measurements of body temperature of EtOH- and oxazolone-treated mice were taken every 30 minutes and followed until 6 hours after treatment with oxazolone. Body temperature is shown as mean ± SEM, as determined by the repeated-measures two-way ANOVA test. ****p < 0.001; (n = 8 mice per group). (D) Bar graphs represent serum levels of HMGB1 in naïve, EtOH- and oxazolone-treated mice 6h post colitis induction as determined by the Mann-Whitney test. Ns, not significant; (n = 3–4 mice per group). (E) Paraffin-embedded colon sections were stained with hematoxylin and eosin (H&E) assessment of tissue alteration. Representative images of colonic sections stained with H&E from control naïve, EtOH- and oxazolone-treated mice at 6 and 24 hours after colitis induction. Scale bars are 40 μm in the upper panel and 100 μm in the lower panel. (F) Th1, chemokines and Th2 (G) gene expression was quantified in colonic tissue isolated from EtOH- and oxazolone-treated mice. Data are expressed as mean ± SEM of fold increase versus naïve mice as determined by the repeated-measures two-way ANOVA test. Ns, not significant; *p < 0.05; (n = 3–6 mice per group).

In contrast to previous studies, we observed a high mortality rate upon oxazolone instillation. Already 24 hours after oxazolone instillation, 37% of mice had died, a percentage further increasing to 67% at 48 hours. Of note, also 25% of the EtOH-treated group died (Fig 1A). These observations led us to better characterize local and systemic responses underlying the early mortality in this model. As oxazolone is described as a hapten allergen, we first monitored intradermal body temperature in the first 6 hours after oxazolone exposure as a read-out for anaphylaxis. Mice challenged with oxazolone indeed showed a striking drop in body temperature from 37.9 ± 1.0°C pre-exposure to 31.6 ± 1.6°C 6 hours post-exposure (Fig 1B). Both vehicle- and oxazolone-induced mice showed an early drop in body temperature of 4°C ± 1.0°C 30 minutes after intracolonic administration, probably due to the anesthesia. Oxazolone-induced mice however showed further reduction of body temperature which was evident between 3 and 6 hours post-exposure (Fig 1B).

Given the increased mortality rate and the sudden decrease in body temperature, we next evaluated serum levels of HMGB1, a protein known as a “necrosis marker” released by damaged or necrotic cells, thereby triggering an inflammatory response. In fact, it is well known that HMGB1 plays a key role in mediating systemic inflammation during sepsis [20] and that high levels correlates with bad prognosis. Although not significant, HMGB1 serum levels were increased in OXA-treated mice compared to EtOH-treated mice 6h post oxazolone challenge (Fig 1C).

Histological analysis of colon sections showed a high degree of tissue damage and inflammation as evidenced by pronounced enterocyte loss, edema and a notable reduction in goblet cell number already 6 hours after oxazolone exposure and an almost complete destruction of the mucosal layer at 24 hours (Fig 1D). Of note, also colon instillation of ethanol led to comparable mucosal damage at 6 and 24 hours, indicating that the acute and severe tissue damage may mainly results from the solvent (ethanol) in which oxazolone is dissolved. As the mortality rate in the EtOH-treated group is however much lower, other mechanisms must contribute to this severe outcome. Assessment of colonic gene expression at 6 and 24 hours post-instillation already showed a strong induction of the pro-inflammatory mediators *il6*, *cxcl1* and *tnfα* at 6 hours in both the EtOH and OXA group (Fig 1E). In contrast, the Th2 cytokines *il4* and *il9* were only increased in OXA-treated mice, but not in EtOH-treated group (Fig 1F). These findings suggest that the combination of a high mortality rate, hypothermia and Th2 cytokine induction is distinctive of the oxazolone-treated mice, in contrast to the early tissue damage caused by ethanol. Moreover, the increased mortality rate was paralleled by increased serum levels of HMGB1, supporting the hypothesis that mortality results from sepsis due to the severe mucosal damage [21].

### Oxazolone treatment triggers influx of NK-T cells in the colonic mucosa

**Fig 2:**
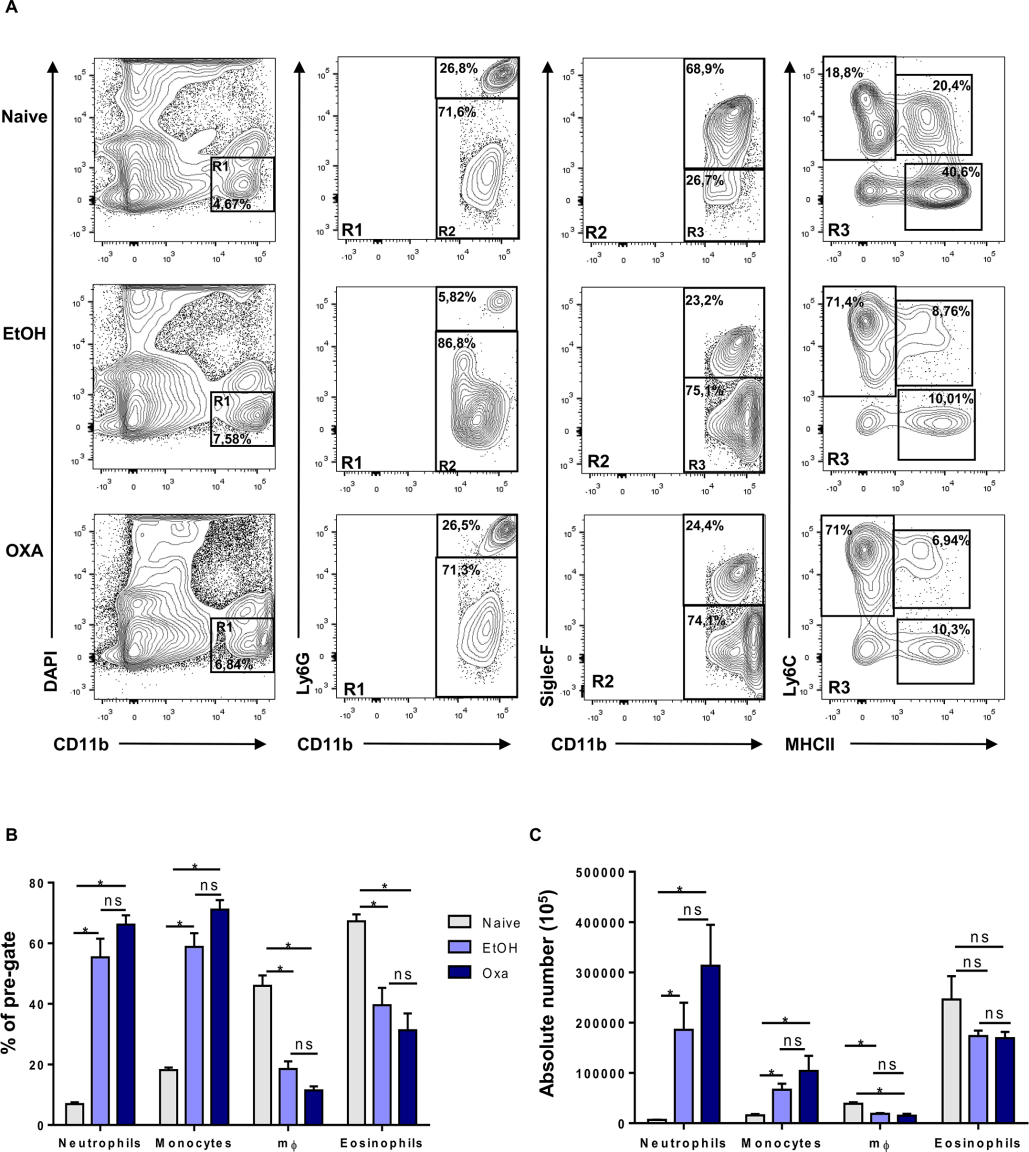
Immune profile of oxazolone-induced colitis. (A) Representative expression of DAPI and CD11b (left column), Ly6G and CD11b (mid-left column), SiglecF and CD11b (mid-right column) and Ly6C and MHCII (right column) from the colon of naïve, EtOH- and oxazolone-treated mice 6 hours post colitis induction. Frequencies (B) and absolute numbers (C) of Ly6G^+^CD11b^+^, Ly6C^+^MHCII^-^ and Ly6C^-^MHCII^+^ and SiglecF^+^CD11b^+^ cells among CD11b^+^, SiglecF^-^CD11b^+^, Ly6G^-^CD11b^+^ cells from colon of naïve, EtOH- and oxazolone-treated mice 6 hours post colitis induction. Data are expressed as mean ± SEM as determined by the Mann-Whitney test. ns, not significant; *p < 0.05; (n = 3–6 mice per group).

To further identify the immune cell populations involved in OXA versus EtOH-induced colitis, flow cytometric immune profiling was performed in naïve, EtOH and OXA-treated mice 6 hours post-induction. Both EtOH and OXA treatment led to a pronounced and significant influx of neutrophils and monocytes to the colonic LP (Fig 2). In addition, OXA and EtOH treatment resulted in a significantly decreased influx of macrophages and eosinophils compared to naïve mice (Fig 2).

**Fig 3:**
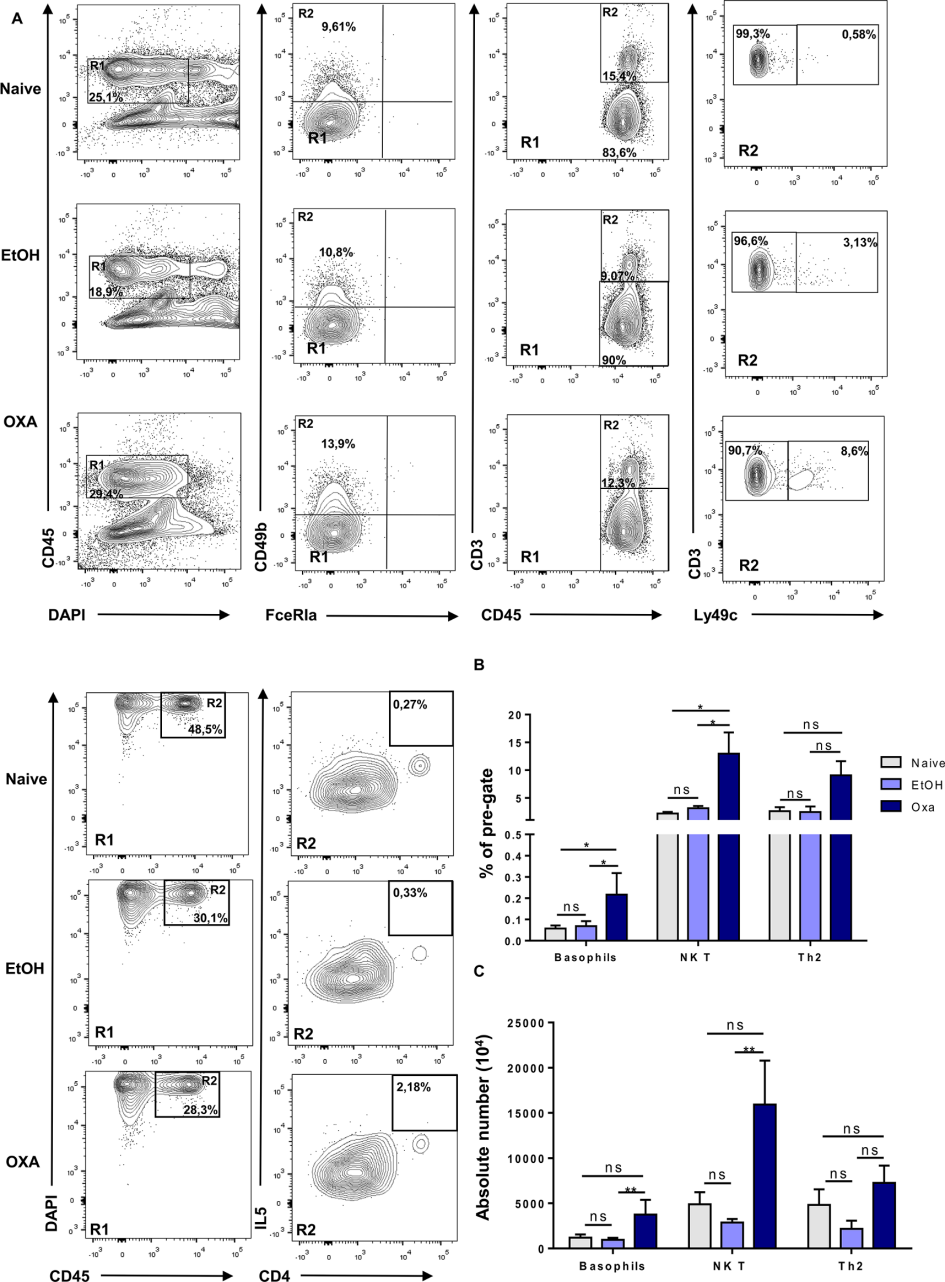
Induction of Th2 and NK T cells is specific to oxazolone-induced colitis treatment. (A) Representative expression of DAPI^-^CD45^+^ cells (left column), CD49b^+^ and FceRIa^+^ cells (mid-left column), CD3^+^CD45^+^ cells (mid-right column) and Ly49c^+^CD3^+^ cells (right column), and DAPI^-^CD45^+^ cells (left column) and CD4^+^IL5^+^ cells (right column) from the colon of naïve, EtOH- and oxazolone-treated mice 6 hours post colitis induction. Frequencies (B) and absolute numbers (C) of CD49b^+^ FceRIa^+^, CD3^+^CD49c^+^, CD4^+^IL5^+^ cells from colon of naïve, EtOH- and oxazolone-treated mice 6 hours post colitis induction. Data are expressed as mean ± SEM as determined by the Mann-Whitney test. Ns, not significant; *p < 0.05; **p < 0.005; (n = 3–6 mice per group).

As we observed a pronounced induction of Th2 cytokines in OXA-treated mice, we next focused on the type of immune cells involved in the Th2 response, including basophils, natural killer T-cells (NKT) and Th2 cells (Fig 3). OXA-treated mice showed a significantly higher proportion of these cell types, a finding that was most evident for CD3^+^ Ly49c^+^ NK T-cells (Fig 3B and 3C).

Taken together, these data clearly show that EtOH and OXA both lead to a strong influx of innate immune cells, including neutrophils and monocytes. In contrast to EtOH, OXA treatment also triggers the influx and/or induction of NK T-cells and, to a lesser extent, Th2 cells, suggesting that these cells may be involved in the higher mortality rate.

### Oxazolone treatment triggers a local and systemic immune response

**Fig 4:**
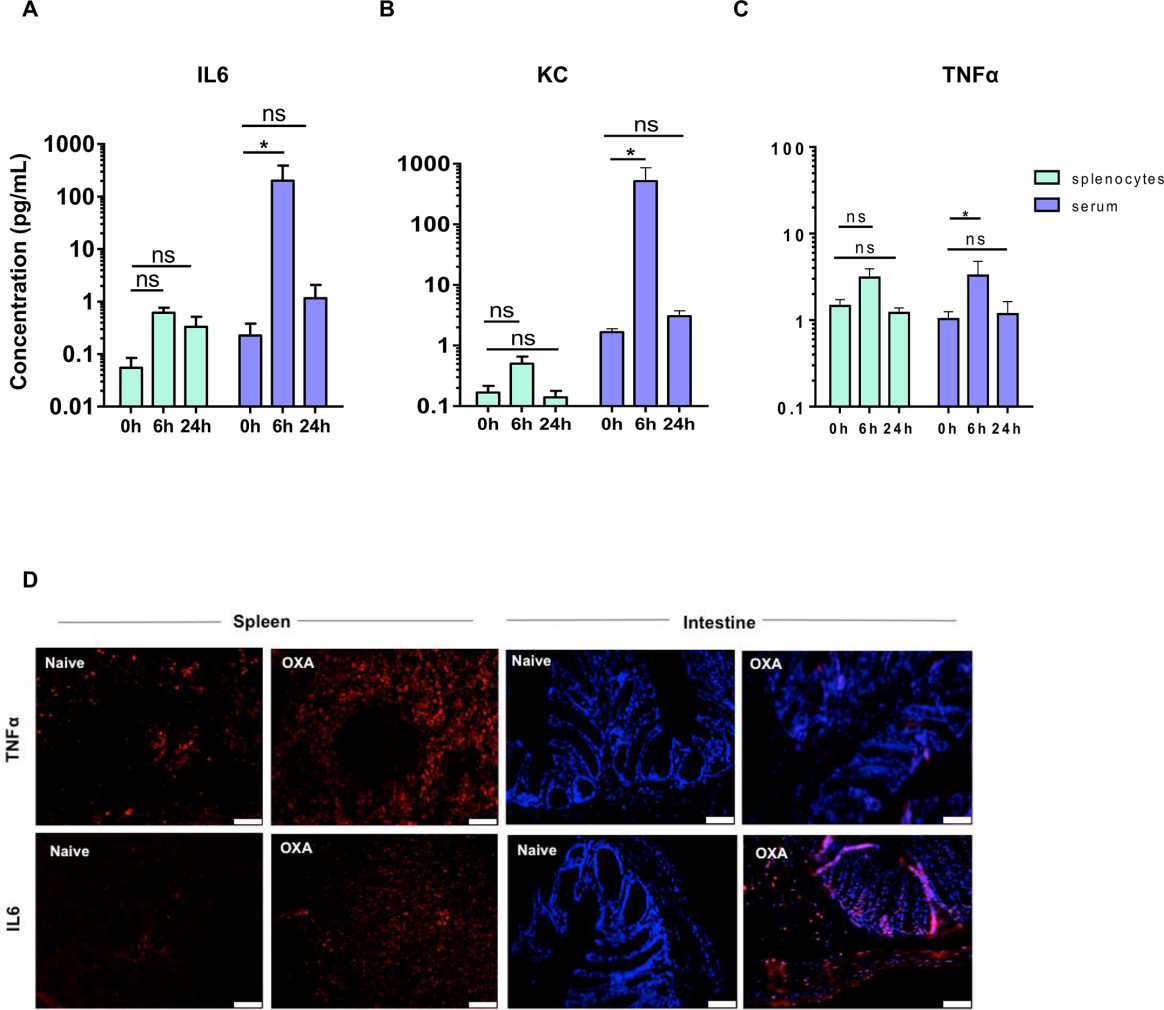
Oxazolone exposure leads to early induction of systemic cytokines. (A-C) Bar graphs represent cytokines in serum and isolated splenocytes of oxazolone-treated mice in both early (6h) and late (24h) phases of the disease. Data are expressed as mean ± SEM as determined by the repeated-measures two-way ANOVA test. ns, not significant; *p < 0.05; (n = 4 mice per group). (D) TNFα and IL6 immunofluorescence on spleen and colon section of naïve and oxazolone-treated mice 6 hours post colitis induction. Scale bars are 40 μm.

We next further characterized the immune response and evaluated the degree of systemic immune activation by measuring serum and spleen cytokine levels. To this end, we analyzed concentrations of TNFα, IL-6 and CXCL1 (Keratinocyte Chemoattractant, KC) in serum and supernatant of cultured splenocytes at different time points. In line with the colonic gene expression data, serum cytokine levels were mainly increased at 6 hours post-oxazolone administration, in particular IL-6 and CXCL1, returning to baseline levels at 24 hours (Fig 4A–4C). Serum TNFα concentration remained relatively low (Fig 4C).

To examine the potential contribution of the spleen, splenocytes were isolated at 0, 6 and 24 hours after OXA or EtOH treatment. Cultured splenocytes however did not produce high levels of IL-6 and CXCL1, suggesting that the colon represents the main source of these circulating cytokines (Fig 4A and 4B). Of note, the kinetics of TNFα serum levels coincided with that of TNFα production by cultured splenocytes, suggesting the spleen as potential source of TNFα. To validate this observation, we performed immunohistochemistry for TNFα and IL-6 on splenic and colonic sections 6 hours after oxazolone exposure, showing increased immunoreactivity for TNFα in the spleen but not in the colon after OXA treatment (Fig 4D). In contrast, increased IL-6 immunoreactivity was observed in both spleen and colon (Fig 4D). These data suggest that oxazolone induces a severe and acute inflammatory response in the colon, associated with a systemic inflammatory response and splenic immune activation.

### Vagal nerve stimulation ameliorates oxazolone-induced colitis and improves survival

Vagus nerve stimulation (VNS) is a well-established and FDA-approved technique to treat drug-resistant epilepsy and depression [22]. Previously, we and others showed that electrical activation of the vagus nerve has anti-inflammatory properties [23] [24]. Electrical stimulation of the efferent VN indeed dampens intestinal resident macrophages and attenuates systemic inflammation mainly by suppressing the release of pro-inflammatory cytokines from splenic macrophages (e.g. TNFα). Hence, we studied to what extent VNS is protective against oxazolone-induced colitis.

**Fig 5:**
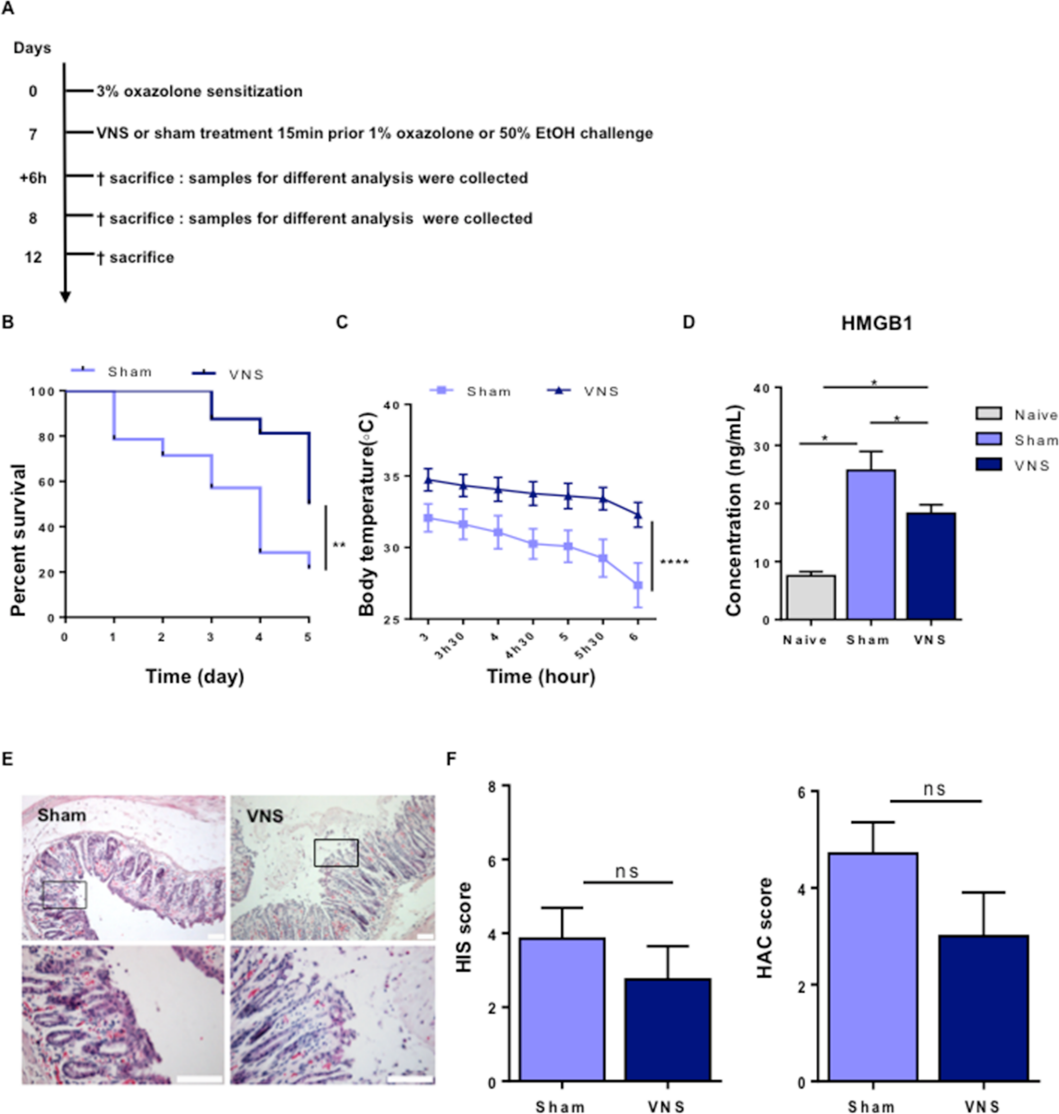
Vagal nerve stimulation improves survival in oxazolone-induced colitis. (A) Wild type BALB/c mice were sensitized by epicutaneous application of 3% oxazolone at a dilution of 4:1 in a mixture of acetone and olive oil (100μl) on day 0, followed by VNS or sham treatment and intracolonic administration of 1% oxazolone dissolved in 50% ethanol (100μl) (or 50% ethanol for control group EtOH-treated mice) on day 7 using an intravenous catheter inserted 3 cm in the colon. Mice were sacrificed 6 hours and 5 days post oxazolone administration and tissue samples were collected for different analysis. (B) Survival proportion was assessed daily. Mortality is expressed as survival rate and shown by Kaplan-Meier survival curves; statistical significance of Kaplan-Meier survival curves was determined with Gehan-Breslow-Wilcoxon test. **p < 0.005; (n = 14–16 mice per group). (C) Repeated measurements of body temperature of EtOH- and oxazolone-treated mice were taken every 30 minutes and followed until 6 hours post colitis induction. Body temperature is shown as mean ± SEM, as determined by the repeated-measures two-way ANOVA test. ****p < 0.001; (n = 7–8 mice per group). (D) Bar graphs represent serum levels of HMGB1 in naïve, EtOH- and oxazolone-treated mice 6 hours post colitis induction as determined by the Mann-Whitney test. *p > 0.05; (n = 2–16 mice per group). (E) Paraffin-embedded colon sections were stained with hematoxylin and eosin (H&E) assessment of tissue alteration. Representative images of colonic sections stained with H&E from control sham and VNS-treated mice 6 hours post colitis induction. Scale bars are 40 μm in the upper panel and 100 μm in the lower panel. (F) Bar graphs represent histological injury score and histological assessment of colitis score of colonic samples of sham and VNS-treated mice 6 hours post colitis induction. Data are expressed as mean ± SEM as determined by the Mann-Whitney test. Ns, not significant; (n = 7–8 mice per group).

VNS-treated mice showed a significant decrease in mortality rate (VNS-treated mice 50% vs sham-treated mice 79%, p = 0.009), together with reduced hypothermia in the early phase (3–6 hours) (Fig 5A and 5B). Previous studies [25] [26] identified HMGB1 as a late mediator of lethal systemic inflammation in sepsis, acting as a pro-inflammatory cytokine inversely related to survival. Therefore, we evaluated the effect of VNS on HMGB1 serum levels 6h post oxazolone administration and VNS application. Of interest, serum HMGB1 levels were significantly reduced by VNS, most likely explaining the improved survival of VNS-treated mice (Fig 5C). In contrast, however, no effect on mucosal damage was observed (Fig 5D and 5E). These results indicate that VNS is able to decrease mortality rate but does not prevent against colonic damage.

**Fig 6:**
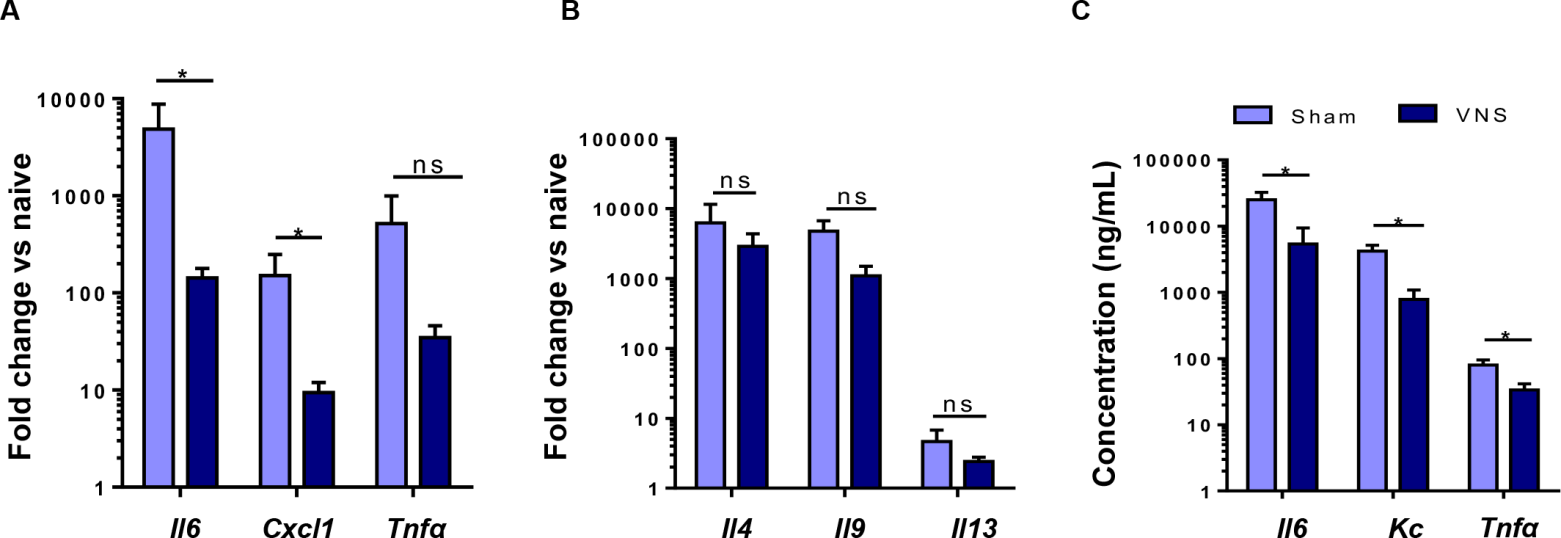
Vagal nerve stimulation reduces colonic and systemic inflammation. (A-B) Bar graphs represent Th1 (A) and Th2 (B) gene expression in colonic tissue isolated from sham- and VNS-treated mice challenged with oxazolone 6 hours after colitis induction. (C) Bar graphs represent serum levels of IL6, KC and TNFα in sham- and VNS-treated mice challenged with oxazolone 6 hours after colitis induction as determined by the Mann-Whitney test. Data are expressed as mean ± SEM; *p < 0.05; ns, not significant; (n = 21–27 mice per group).

Given the pronounced effect of VNS on survival rate and body temperature, we next evaluated the effect of VNS on colonic and serum cytokines. In the colon, VNS led to a significant reduction in *il6* and *cxcl1* gene expression, there was a tendency for reduction in *Tnfa* (Fig 6A). However, no differences were detected in *il4*, *il9 and il13* gene expression (Fig 6B). In line, VNS significantly reduced serum levels of IL-6, CXCL1 and TNFα (Fig 6C). Together, these data support the idea that VNS attenuates HMGB1 levels and affects the colonic immune response, mainly reducing IL-6 and CXCL1 production in oxazolone-induced colitis eventually leading to an improved survival.

## Discussion

Although oxazolone-induced colitis has been used repeatedly in the literature as a model of IBD, insights into the underlying immune response are surprisingly very limited. In the present study, we therefore first characterized the immune response and clinical parameters in this model. We demonstrated that the hapten oxazolone triggers a Th2-mediated immune response, associated with a high mortality rate, rapid development of hypothermia and severe mucosal damage. Next, we showed that VNS attenuates both colonic and systemic cytokines and improves survival, most likely by reducing HMGB1 levels. Although the severity of mucosal damage, partly due to ethanol, was not affected by VNS, these data further support the anti-inflammatory properties of VNS, even in an aggressive model as oxazolone-induced colitis, and warrant further study of this technique as potential new treatment of acute inflammation.

The initial aim of this study was to acquire further insight into the kinetics and pathological features of oxazolone-induced colitis, a pre-clinical model of UC mainly affecting the distal part of the colon. Deeper understanding into this model was essential to define disease-specific parameters and to evaluate the therapeutic and translational feasibility of vagus nerve stimulation for the treatment of UC. Oxazolone-induced colitis was proposed almost 20 years ago as a pre-clinical model for UC, mainly supported by the presence of a typical type 2 (Th2) immune response [12]. Although we were able to confirm the presence of a Th2 response upon oxazolone instillation, several other pathological features were not in line with what is seen in UC. First, we observed that colonic instillation of oxazolone leads to an early and severe decline in body temperature and a high mortality rate of 67% at 48 hours after oxazole instillation. Although mortality rates of 20 and 40% have been reported previously, we have no explanation for this observation and can only speculate that differences in the microbiome may have contributed [27, 28]. Second, whereas UC is mostly characterized by superficial inflammation of the mucosa, oxazolone exposure leads to a rapid and complete destruction of the epithelial barrier. These findings challenge the idea that oxazolone colitis represents a suitable model for UC and suggests that it rather is comparable to sepsis. At best, it may resemble a very severe type of UC with complete mucosal loss, as observed in patients with toxic megacolon. The significant decrease in body temperature, increase in serum and splenic cytokines and in particular the high mortality rate associated with an increase in serum HMGB1 levels, would rather indicate that these mice develop sepsis. Of note, Nolte et al. previously claimed that oxazolone colitis represents a model of sepsis, [29] further underscoring the need to revisit this model. Another interesting and so far largely neglected finding, is the mucosal damage evoked by ethanol, the solvent of oxazolone. Of note, colonic instillation of ethanol leads to a similar destruction of the mucosal barrier as seen in OXA-treated mice, leading to increased pro-inflammatory gene expression and influx of monocytes, neutrophils and basophils. In contrast to oxazolone however, the drop in body temperature and lethality were less pronounced, indirectly suggesting that the Th2 response induced by oxazolone most likely contributes to the more severe disease course observed. Taken together, our findings question the suitability of oxazolone-induced colitis model as a model of UC, given its very aggressive disease course.

In 2000 the group of Tracey for the first time reported that stimulation of the vagus nerve attenuates systemic inflammation and improves survival in a model of sepsis [24]. This anti-inflammatory effect of VNS requires an intact splenic nerve [30] and alpha 7 nicotinic receptor (α7nAChR) expression on splenic macrophages. Over the years, the anti-inflammatory properties of VNS have also been reported in a variety of other inflammatory animal models, including models of intestinal inflammation such as postoperative ileus [13] [23] [31] and colitis [32] [33]. Electrical and pharmacological activation of the vagal anti-inflammatory pathway has been previously studied as a novel approach to treat IBD in several animal models. In a rat model of TNBS colitis, the group of Bonaz showed that 5 days of VNS prevented body weight loss and improved colon mucosal damage. This effect was associated with a decrease in TNFα, IL-1β and IL-6 levels via interference with the JAK2/STAT3 signaling pathway. [5] Activation of vagal efferents can also be achieved by pharmacological activation of the motor nucleus of the vagus nerve in the brainstem. Indeed, central activation of muscarinic acetylcholine 1 receptors and blockade of acetylcholine esterase mimic the effect of VNS in sepsis. [34] [35] Also in DSS colitis, central activation of the vagus nerve by galantamine, an acetylcholinesterase inhibitor, reduces the severity of DSS-induced colitis in mice. [4] Conversely, vagotomy increases the susceptibility to develop DSS colitis, [36] [37] [38] further supporting the immunomodulatory role of the cholinergic anti-inflammatory pathway in Th1/Th17 mediated colitis.

In this study, we evaluated to what extent VNS may also be applied to improve Th2-mediated colitis. Even though it is generally accepted that the VN densely innervates the gastrointestinal tract, vagal innervation declines in density along the intestine and fails to innervate the distal colon. In previous studies by Berthoud HR et al. [39, 40] it has been shown that efferent vagal nerve terminals directly synapse with postganglionic neurons located in the enteric nervous system, which might propagate the beneficial effect of the stimulation. In fact, as in TNBS-induced colitis, VNS reduced the serum levels of pro-inflammatory cytokines, i.e. IL-6, CXCL1, and TNFα, and dampened the colonic expression of IL-6 and CXCL1 in the oxazolone-induced colitis model. However, no reduction in colonic expression of Th2 cytokines could be demonstrated, at least not at 6 hours after oxazolone administration. Of note, although these data suggest a beneficial effect of VNS on colonic inflammation, no improvement in mucosal damage could be detected. This may be explained by the severity of the damage induced, which is mediated to a large extent by the caustic effect of ethanol, and will thus not be affected by VNS. Moreover, it has to be taken into account that severe colonic lesions, as the one observed in oxazolone-induced colitis, requires long time to heal. This might have occurred in case mice would have survived the disease and lived long enough. Studies in experimental model of CD (5) and findings in patients suffering from CD (6) have demonstrated that VNS does not heal colonic lesion in short time (e.g. 6 hours post VNS treatment).

Nevertheless, the anti-inflammatory effect of VNS proved to be effective even in such a severe model of colitis resulting in a significant improvement of survival compared to sham-treated mice, a finding associated with a reduction in HMGB1 serum levels. As the beneficial effect of VNS on colonic inflammation was only mild, increased survival most likely results from its effect on HMGB1 production, a key determinant of survival in sepsis [21]. HMGB1 is an intracellular protein that, when secreted in the extracellular compartment, becomes a powerful mediator of systemic inflammation functioning as a cytokine that enhances both innate and adaptive immune responses. It has been widely studied in rodent sepsis models and has been identified as a late mediator of lethal systemic inflammation released by macrophages after stimulation with endotoxin, TNF, or IL-1 [41]. Damage to the mucosal barrier induced by ethanol and oxazolone will trigger the release of intracellular danger signals, including HMGB1. Moreover, absence of the intestinal mucosa will allow massive entry of micro-organisms into the systemic circulation, ultimately leading to sepsis and death. Of note, the beneficial effect of VNS in sepsis has been attributes to suppression of HMGB1, an effect mediated by acetylcholine alpha7 nicotinic acetylcholine receptor activation on macrophages [25]. Of interest, we here showed that HMGB1 was also increased in oxazolone-treated mice, while VNS significantly reduced serum levels of HMGB1 in oxazolone-treated mice compared to control. These data suggest that the increased survival rate in VNS-treated mice can most likely be explained by the effect of VNS on HMGB1 release.

In conclusion, our study sheds new light on the oxazolone-induced colitis model, providing for the first time a critical characterization of this pre-clinical model of UC. It should, however, be noted that even though no single animal model entirely mimics the clinical and histopathological characteristics of human IBD, the oxazolone-induced colitis model recapitulates rather a model of septic shock, and at best a very aggressive form of colitis. Recently, clinical trials have investigated the possible beneficial effect of VNS in patients with rheumatoid arthritis, postoperative ileus and Crohn’s disease [7, 42, 43] (NCT01552941).
